# Vitamin C Compound Mixtures Prevent Ozone-Induced Oxidative Damage in Human Keratinocytes as Initial Assessment of Pollution Protection

**DOI:** 10.1371/journal.pone.0131097

**Published:** 2015-08-13

**Authors:** Giuseppe Valacchi, Claudia Sticozzi, Giuseppe Belmonte, Franco Cervellati, Julien Demaude, Nannan Chen, Yevgeniy Krol, Christian Oresajo

**Affiliations:** 1 Department of Life Science and Biotechnologies, University of Ferrara, Via L. Borsari, Ferrara, Italy; 2 Department of Food and Function, Kyung Hee University, Seoul, South Korea; 3 L’Oreal Research and Innovation, Clark, New Jersey, United States of America; 4 Skinceuticals, Inc., New York, New York, United States of America; 5 L’Oreal Research & Innovation, Paris, France; University of Sassari, ITALY

## Abstract

**Introduction:**

One of the main functions of cutaneous tissues is to protect our body from the outdoor insults. Ozone (O_3_) is among the most toxic stressors to which we are continuously exposed and because of its critical location, the skin is one of the most susceptible tissues to the oxidative damaging effect of O_3_. O_3_ is not able to penetrate the skin, and although it is not a radical per se, the damage is mainly a consequence of its ability to induce oxidative stress via the formation of lipid peroxidation products.

**Aim of Study:**

In this study we investigated the protective effect of defined “antioxidant” mixtures against O_3_ induced oxidative stress damage in human keratinocytes and understand their underlying mechanism of action.

**Results:**

Results showed that the mixtures tested were able to protect human keratinocytes from O_3_-induced cytotoxicity, inhibition of cellular proliferation, decrease the formation of HNE protein adducts, ROS, and carbonyls levels. Furthermore, we have observed the decreased activation of the redox sensitive transcription factor NF-kB, which is involved in transcribing pro-inflammatory cytokines and therefore constitutes one of the main players associated with O_3_ induced skin inflammation. Cells exposed to O_3_ demonstrated a dose dependent increase in p65 subunit nuclear expression as a marker of NF-kB activation, while pre-treatment with the mixtures abolished NF-kB nuclear translocation. In addition, a significant activation of Nrf2 in keratinocytes treated with the mixtures was also observed.

**Conclusion:**

Overall this study was able to demonstrate a protective effect of the tested compounds versus O_3-_induced cell damage in human keratinocytes. Pre-treatment with the tested compounds significantly reduced the oxidative damage induced by O_3_ exposure and this protective effect was correlated to the abolishment of NF-kB nuclear translocation, as well as activation of Nrf2 nuclear translocation activating the downstream defence enzymes involved in cellular detoxification process.

## Introduction

Epidemiological studies show an increasing trend of environmental changes such as higher temperature, higher incidence of UV radiation, in combination with higher concentrations of pollutants such as carbon dioxide (CO_2_) and nitrogen dioxide (NO_2_) derived from vehicle emissions [1]. In turn, these changes lead to an increase in the tropospheric O_3_ concentrations, which is expected to rise by 5 fold at the end of this century. In fact, O_3_ formation underlies complex interactions, depending on the presence of precursors [nitrogen oxides (NOx) and volatile organic compounds (VOCs)], degrading substances, temperature, and UV-radiation [2]. Typical levels of O_3_ recorded in urban environments can range from 0.2 to 1.2 ppm [3]. Being most exposed, one of the tissues highly susceptible to the deleterious effects of O_3_ is the skin, especially during smoggy and O_3-_alert days [3, 4, 5].

O_3_ is a highly reactive oxidant capable of forming peroxides, aldehydes, and lipid ozonation products (LOP) as a result of unsaturated fatty acid oxidation in biological systems [6, 7, 8] and is known to damage the barrier function of epidermis [8]. The toxicity of O_3_ is largely due to interaction with unsaturated lipids that generate radical products [9] and to the depletion of cutaneous antioxidants [10]. O_3_ exposure not only affects antioxidant levels and oxidation markers in the outermost layer [5, 11] but also induces a cascade of cellular stress responses in deeper cellular layers of the skin [12].

Although the skin is well equipped with enzymatic (glutathione peroxidase, superoxide dismutase and catalase) and a non-enzymatic low molecular weight antioxidant defense system, (vitamin E, vitamin C, glutathione (GSH), uric acid, etc) [13] chronic exposure to environmental stressors can overwhelm the skin’s defensive system and induce persistent damage to cutaneous tissues. Therefore the use of antioxidant supplements as a defensive approach against pollution generated oxidative stress has been suggested, but remains controversial because the offered protection is limited by the first-pass metabolism and the ability to sustain a substantial concentration of antioxidants in the skin. Furthermore, topical use of a single antioxidant molecule will not be able to protect the skin a comprehensive manner. For this reason, the use of a topical cocktail of synergistic antioxidants is a good strategy to overcome these limitations and provide a meaningful benefit. Specifically, it has been demonstrated that the addition of ferulic acid to a topical vitamin C and E solution stabilized and doubled their protection from environmentally induced-oxidative stress [14,15]. On the basis of these findings, researchers have been encouraged to explore new ways for preventing or neutralizing the toxic effects of O_3_ in cutaneous tissue. In the present study, we investigated the protective effect of pure topical antioxidant mixtures against O_3-_induced oxidative stress damage in human keratinocytes.

## Materials and Methods

### Cells

Normal human epidermal keratinocytes (NHEK) (Clonetic; BioWittaker, Wokingham, Berks.) were cultured in Keratinocyte Growth Medium (Clonetic; BioWittaker, Wokingham, Berks.) with 0.06 mM calcium containing 100 μ/ml penicillin, 100 μ/ml streptomycin, and 10% fetal bovine serum (Lonza, Milan, Italy) and incubated at 37°C in 5% CO_2_.

### Antioxidant Mixtures

Prior to O_3_ exposure, cells were pre-treated for 24 hrs with the following mixtures:

MIX 1: 15% L-ascorbic acid + 1% Alpha-tocopherol + 0.5% Ferulic Acid
MIX 2: 10% L-ascorbic acid + 2% Phloretin + 0.5% Ferulic Acid

Control cells were not treated with the mixtures and only exposed to filtered air.

### O_3_ exposure

O_3_ was generated from O_2_ by electrical corona arc discharge (ECO_3_ model CUV-01, Torino, Italy). The O_2_–O_3_ mixture (95% O2, 5% O_3_) was combined with ambient air and allowed to flow into a Teflon-lined exposure chamber, with the O_3_ concentration in chamber adjusted to varying ppm outputs and continuously monitored by an O_3_ detector. Exposure to filtered air was carried out in similar exposure chambers except that filtered airflow was released into the chamber at flow rates similar to the O_3_ output. After pre-treatment with different antioxidant mixtures, cells (1x10^6^ cells/well in 1.2 ml of media in 6 cm Petri dishes) were exposed to filtered air or different O_3_ concentrations (0.1, 0.2 or 0.5 ppm) for 30 min or 1 h.

Subsequently, the medium was replaced with fresh medium + 10% FBS (3 ml). The O_3_ dose and the exposure time were determined by the current literature on O_3_ pollution levels. Temperature and humidity were monitored during exposures (37°C and 45–55%, respectively).

### Cellular viability

Viability studies were performed 24 h after O_3_ treatment by measurement of LDH release and cytofluorimetric assay as previous described [16]. The LDH levels in the supernatant were calculated base on the kit instructions (EuroClone Milan, Italy). All tests were performed in triplicate and assays were repeated five times independently with average results reported.

Cytofluorimetric assay was performed using Muse Count & Viability Kit (Millipore, Corporation, Billerica, MA, USA). Briefly, cells (1x10^6^ to 1x10^7^ cells/ml) were suspended in PBS. Then, 380 μl of Muse Count & Viability working solution was added to the cells, and 20 μl of this cell suspension was incubated for 5 minutes at room temperature in the dark. Cells were analyzed by using a Muse Cell Analyzer.

### Cellular Proliferation

Keratinocytes proliferation was determined by the BrdU Cell Proliferation Assay Kit which detects 5-bromo-2’-deoxyuridine (BrdU) incorporation in the DNA of proliferating cells as previously described [17]. Briefly, cells were cultured in a labelling medium that contains BrdU, (pyrimidine analog) which is incorporated into the newly synthesized DNA of proliferating cells. After washing, the cells were fixed and the DNA denatured and then BrdU mouse Ab was added. Anti-mouse IgG, HRP linked antibody, and the TMB substrate were added and the colorimetric reaction was developed.

### Protein carbonyls

Carbonyl groups in proteins were determined by OxyBlot (Chemicon, USA). Briefly, after derivatization of carbonyl groups to dinitrophenylhydrazone (DNP-hydrazone) by reacting with dinitrophenylhydrazine (DNPH), the DNP-derivatized protein samples were separated by polyacrylamide gel electrophoresis followed by Western blotting and served as an indicator of oxidative stress.

### Western blotting analysis

Western blotting analysis was performed as previous described [18]. After treatment the cells were seeded (5 × 10^6^ cells/ml) in 100 mm dishes washed twice with ice-cold PBS and then scraped with PBS. 40 μg protein were loaded onto 10% sodium dodecyl sulphate–polyacrylamide electrophoresis gels and then transferred onto nitrocellulose membranes. Blots were blocked in Tris-buffered saline (pH 7.5), containing 0.1% Tween 20 and 3% milk for 1h. Membranes were incubated overnight at 4°C with the appropriate primary antibody and then incubated with horseradish peroxidase-conjugated secondary antibody and the intensity of the chmiluminescence detected (BioRad, Milan, Italy). The blots were then stripped and re-probed with β-actin as the loading control. Images of the bands were digitized and the densitometry of the bands was performed using Image J software.

### DCFH-DA assay

NHEK cells (5 x 10^4^ cell/ml) were incubated with 20 μM DCFH-DA in the loading medium in 5% CO_2_/95% air at 37°C for 30 min. After this time, cells were exposed to O_3_ for different times (15, 30, and 50 minutes) and the fluorescence of the cells from each well was measured at 485 nm (excitation filter) and 530 nm (emission filter) by using a plate reader (TECAN- infinite M200).

### Immunocytochemistry

Human keratinocytes were grown on coverslips at a density of 1 × 10^5^ cells/ml, and after treatment fixed in 4% paraformaldehyde for 30 min at room temperature as previously described [17]. Cells were permeabilized for 5 min at room temperature with PBS containing 0.2% Triton X-100, then the coverslips were blocked in PBS containing 1% BSA at room temperature for 1h. Coverslips were then incubated with primary antibody in PBS containing 0.5% BSA at 4°C overnight. After washing, coverslips were incubated with appropriate secondary antibody for 1 h at room temperature. Nuclei were stained with 1 μg/ml DAPI (Sigma- Aldrich) for 1 min. Coverslips were mounted onto glass slides using anti-fade mounting medium 1,4 diazabicyclooctane (DABCO) in glycerine and examined by the Leica light microscope equipped with epifluorescence at × 630 magnification. Negative controls for the immunostaining experiments were performed by omitting primary antibodies. Images were acquired and analyzed with Leica software.

### Quantitative real-time PCR

Quantitative real-time PCR was carried out as described in detail previously [18]. Briefly, total RNA was extracted, using an AURUM total RNA Mini Kit with DNase digestion (Bio-Rad), from 2×10^5^ keratinocytes for each experimental condition, according to the manufacturer’s recommended procedure. First-strand cDNA was generated from 1 μg of total RNA using the iScript cDNA Synthesis Kit (Bio-Rad). The primer pairs (Table 1) capable of hybridization with unique regions of the appropriate gene sequence were obtained from the Real-Time PCR GenBank Primer and Probe Database Primer Bank, RTPrimerDB. Quantitative real-time PCR (qPCR) was performed using SYBR green on the CFX Multicolor real-time PCR detection system (Bio-Rad). The final reaction mixture contained 300 nM each primer, 1 μl of cDNA, and 7 μl of iQ SYBR Green Supermix (Bio-Rad), with RNase-free water being used to bring the reaction mixture volume to 15 μl. All reactions were run in triplicate. Real-time PCR was initiated with a 3-min hot-start denaturation step at 95°C and then performed for 40 cycles at 95°C for 3 s and 60°C for 5 s. During the reaction, fluorescence, and therefore the quantity of PCR products, was continuously monitored by Bio Rad CFX Manager software (Bio-Rad). Primers were initially used to generate a standard curve over a large dynamic range of starting cDNA quantities, permitting calculation of the amplification efficiency (a critical value for the correct quantification of expression data) for each of the primer pairs. Ribosomal proteins L13a (RPL13a) and L11a (RPL11a) and GAPDH were employed as reference genes. Samples were compared using the relative cycle threshold (CT). After normalization to more stable mRNA RPL13a, RPL11a, and GAPDH, the fold increase or decrease was determined with respect to control, using the formula 2−ΔΔCT, where ΔCT is (gene of interest CT) (reference gene CT), and ΔΔCT is (ΔCT experimental)(ΔCT control).

**Table 1:** Primer sequences and PCR condition.

Gene	Primer sequence	T_a_ °C	Product length (bp)	QPCR Amplification Efficiency (%)	n° of cycles	Ref. Primer Bank
**IL-8**	F: 5'- ggtgcagttttgccaaggag-3' R: 5'- ttccttggggtccagacaga -3'	59.9	183	98.6	39	GenBank Accession NM 000584.3
**RPL13A**	F: 5'-cctaagatgagcgcaagttgaa- 3' R: 5'-ccacaggactagaacacctgctaa-3'	60.2	203	97.3	39	Pattyn *et al*. 2006
**RPL11A**	F: 5'- tgcgggaacttcgcatccgc-3' R: 5'- gggtctgccctgtgagctgc-3'	60.1	108	96.5	39	GenBank Accession NM 000975.2
**GAPDH**	F: 5'- tgacgctggggctggcattg-3' R: 5'- ggctggtggtccaggggtct -3'	60	134	94.6	39	GenBank Accession NM 002046.3

Data calculated by Bio-Rad CFX Manager Software (Bio-Rad).

### Statistical Analysis

Two-way analysis of variance (ANOVA) test was used for each of the tested variables. Results were considered significant with a *P*-value<0.05. Data are expressed as mean ± S.D. of triplicate determinations obtained in 5 independent experiments.

## Results

### Effect of antioxidant mixtures on cytotoxicity induced by O_3_ exposure

**Fig 1:**
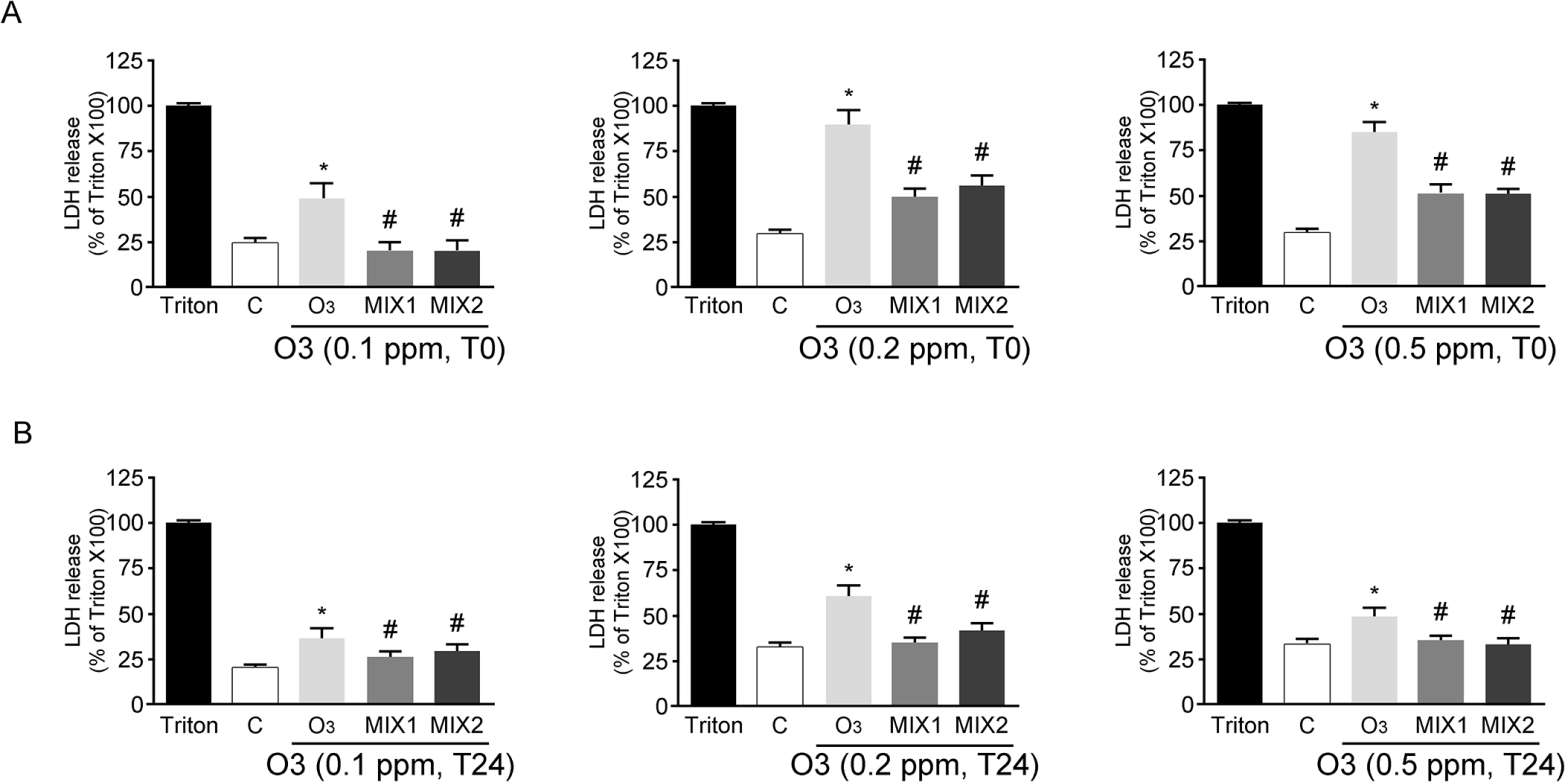
Cytotoxicity measured by using LDH release at T0 (A) and T24 (B) in human keratinocytes exposed to O_3_ pre-treated with/without MIXs. Triton X represents 100% of LDH release. Data are expressed as percentage of Triton X-100 (averages of five experiments ± SEM, *p < 0.05 vs control; #*p* < 0.05 vs O3).

The first set of experiments evaluated the protective effect of the antioxidant mixtures versus O_3_ exposure. As depicted in the Fig 1A, the cells exposed to different concentration of O_3_ (0.1, 0.2 or 0.5 ppm) for 1 hr (T0) showed an increased LDH release, while pre-treatment with the mixtures for 24 hrs prevented this effect. 24 hrs post-O_3_ exposure (T24) the LDH levels were still higher than the control (although lower than at T0) and the protective effect of the mixture pre-treatment was still significant (Fig 1B).

### Effect of antioxidant mixtures on decrease in cell proliferation induced by O_3_ exposure

**Fig 2:**
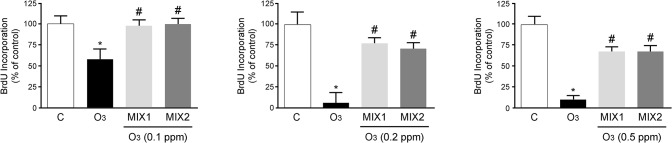
MIX 1 and MIX 2 pre-treatment prevents the decrease in cell proliferation 24 hr after O_3_ exposure. Data are expressed as percentage of control (averages of five experiments ± SEM, *p < 0.05 vs control; #*p* < 0.05 vs O_3_.

The next step, by BrdU assay, was to evaluate the effect on cellular proliferation 24 h after O_3_ exposure. As showed in Fig 2, O_3_ negatively affected cell proliferation at 0.1, 0.2, and 0.5 ppm (decrease by 40%, 90% and 85%, respectively) and pre-treatment with the mixtures attenuated this effect. Following experiments were conducted exclusively at 0.1 and 0.2 ppm, because 0.5 ppm was determined too toxic and did not potentiate a dose dependent response.

### Effect of antioxidant mixtures on HNE adduct formation induced by O_3_ exposure

**Fig 3:**
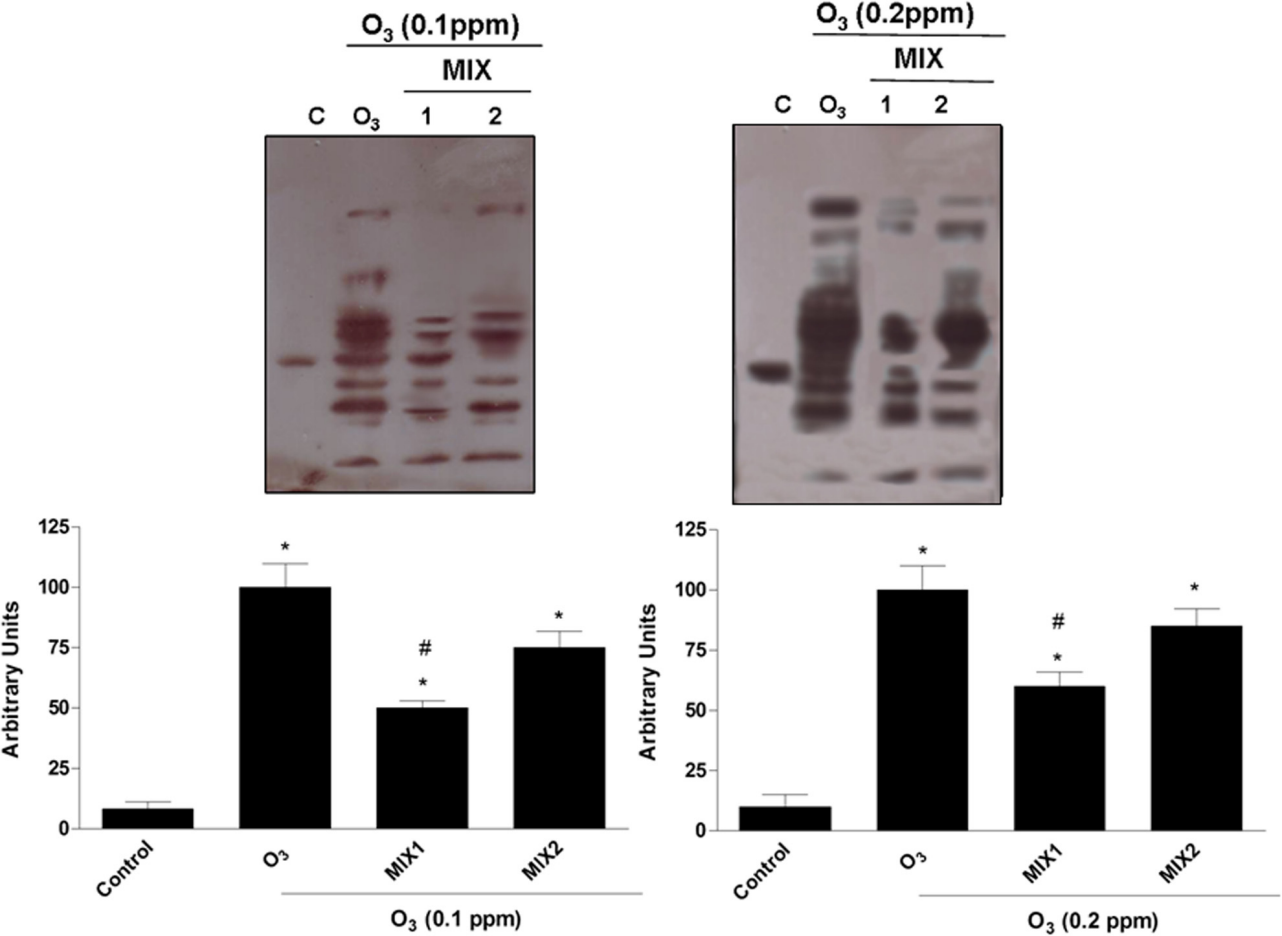
Protective effect of MIX 1 and MIX 2 against O_3_-induced HNE protein adducts formation in human keratinocytes. Representative Western blot is depicted in the top of the figure Quantification of the HNE bands (bottom). Data are expressed in arbitrary units (averages of five experiments ± SEM, *p < 0.05 vs control; #*p* < 0.05 vs O_3_).

A known consequence of O_3_ exposure is the induction of lipid peroxidation with the formation of alpha-beta unsaturated aldehydes such as 4-hydroxy 2-nonenal (HNE) [19]. Because of the ability of HNE to form adducts with target proteins, most cellular proteins can be modified and their functions corrupted. Therefore, we evaluated the protective role of the mixtures against O_3_-induced HNE protein adducts 1 hr after O_3_ exposure (T0). As shown in Fig 3, after O_3_ exposure there was a significant dose dependent increase in HNE protein adduct levels and the pre-treatment with the mixtures (MIX 1 left panel; MIX 2 right panel) clearly prevented HNE protein adducts formation. In addition, pre-treatment with MIX 1 appeared to be more effective respect to MIX 2.

### Pre-treatment with the mixtures decreased carbonyls formation induced by O_3_ exposure

**Fig 4:**
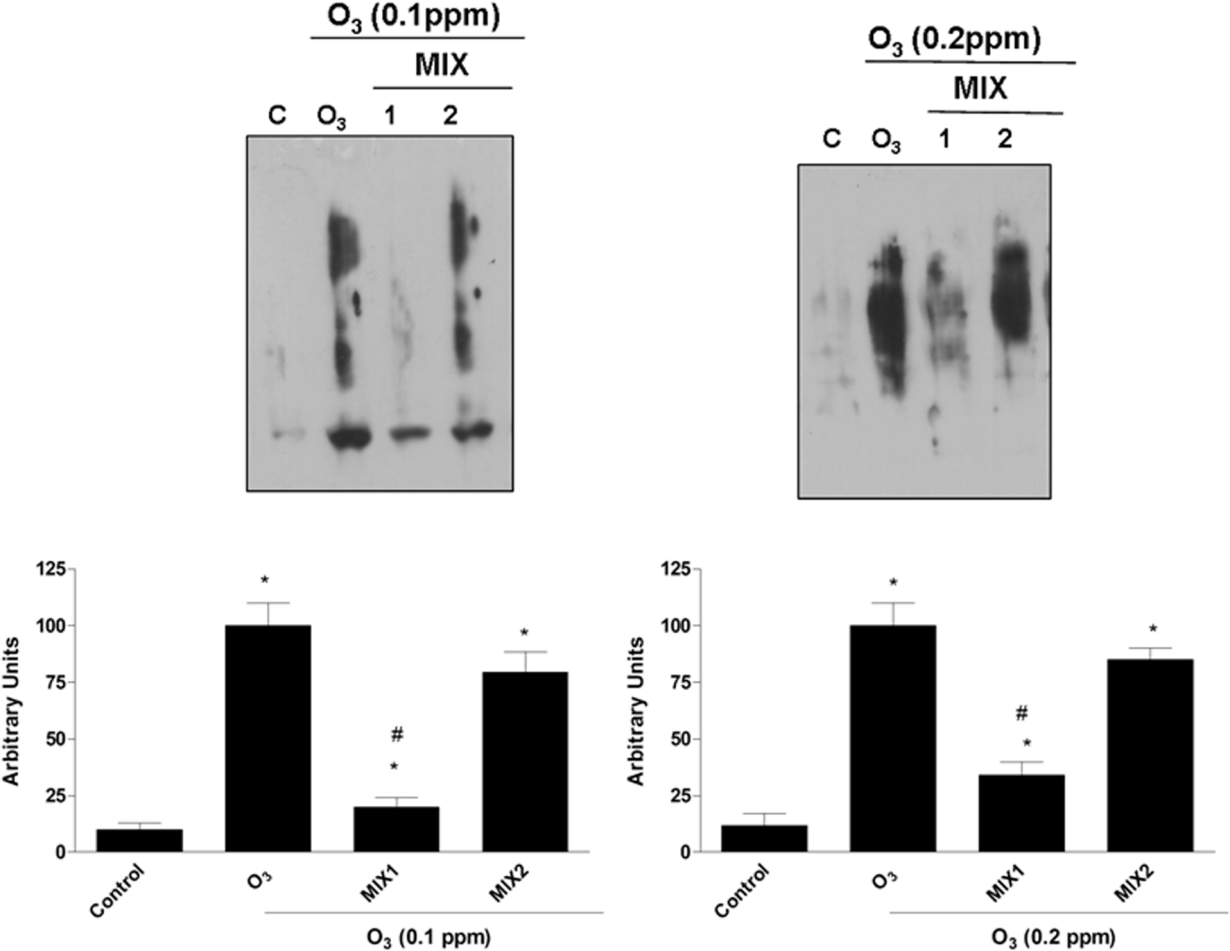
O_3_ induced carbonyl groups formation in human keratinocytes and MIX 1 and MIX 2 pre-treatment prevented this effect. Representative Western blot (top). Quantification of the carbonyl group bands (bottom). Data are expressed in arbitrary units (averages of five experiments ± SEM, *p < 0.05 vs control; #*p* < 0.05 vs O_3_).

To further verify the mixtures ability to protect keratinocytes from O_3_-induced oxidative damage, the levels of carbonyl group protein adducts were also analyzed. As shown in Fig 4, exposure to O_3_ (0.1 ppm) led to a significant increase in carbonyls formation levels and pre-treatment with the mixtures prevented this effect. Same trend was noticed when the cells were exposed to 0.2 ppm O_3_. In the latter case, the level of protein carbonyl groups were higher than that produced by exposure to 0.1 ppm O_3_, indicating a dose-dependent trend.

### Mixture pre-treatment modulates ROS production induced by O_3_ exposure

**Fig 5:**
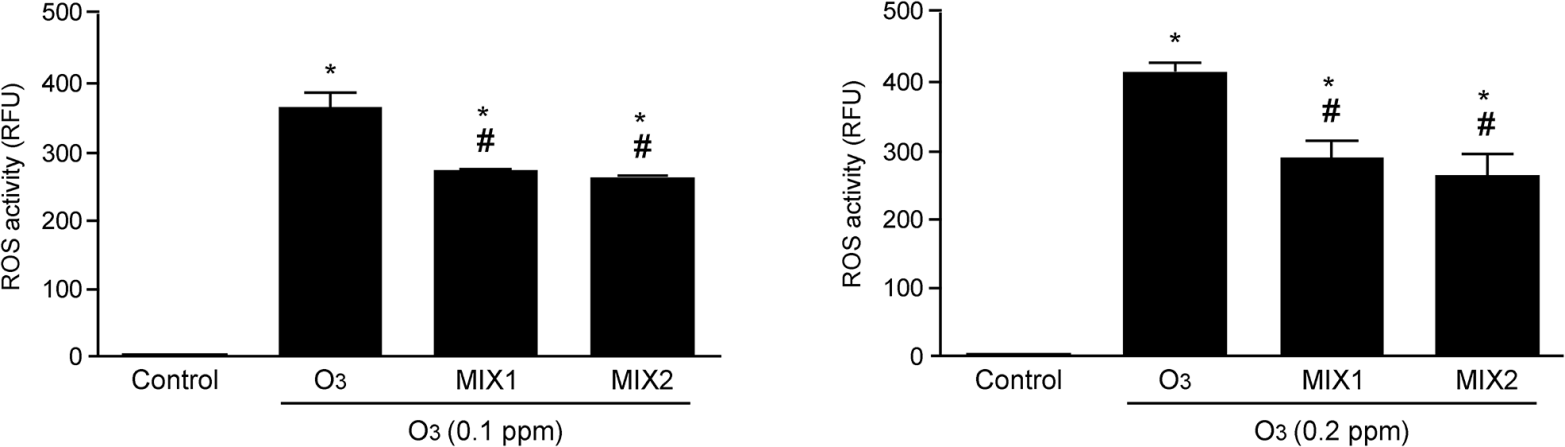
O_3_ induced ROS formation in human keratinocytes and MIX 1 and MIX 2 pre-treatment prevented this effect. ROS production was measured by fluorimetry with DCFH-DA staining. Data are expressed in RFU (averages of five experiments ± SEM, *p < 0.05 vs control; #*p* < 0.05 vs O3).

To confirm the ability of O_3_ exposure to induce ROS formation, we performed the DCFH-DA assay. As showed in Fig 5, ROS levels were increased in a dose dependent manner when the cells were exposed to O_3_ (0.1 and 0.2 ppm) and pre-treatment with the mixtures significantly decreased the ROS formation. Both MIX 1 and MIX 2 were equally effective in reducing ROS.

### Antioxidant effect of different mixtures on activation of NRF-2 pathway induced by O_3_ exposure

**Fig 6:**
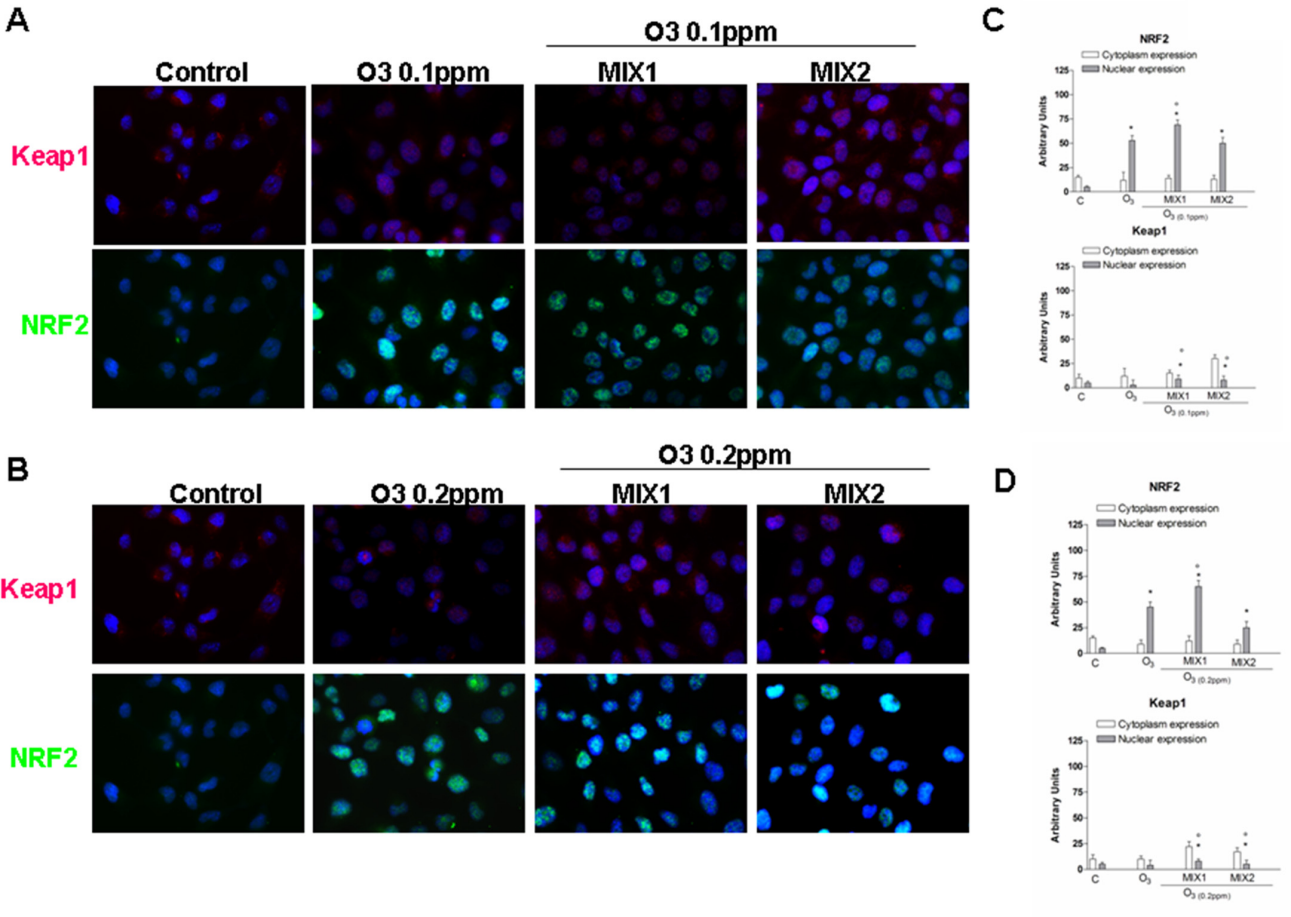
O_3_ induced NRF2 activation in human keratinocytes and MIX 1 and MIX 2 pre-treatment for 24 h potentiated this effect. Immuno-cytochemistry of keratinocytes showing localization of NRF2 (red) and Keap1 after O_3_ exposure for 1 h. Images are merged and representative of at least 100 cells viewed in each experiments (n = 5). Nuclei (blue) were stained with DAPI. Original magnification X 630. Immunoreactivity of NRF2 and Keap1 was semi-quantified as area of both signals into nucleus respect to cytoplasm, by using Image J sophtware. Data are expressed in arbitrary units (averages of five experiments ± SEM, *p < 0.05 vs C (nuclear expression); °*p* < 0.05 vs C (cytoplasm expression)).

To better elucidate the mechanism involved in the protective effects of the MIXs, we evaluated their ability to activate the nuclear transcription factor (erythroid-derived2)-like2 (NRF-2) [20]. Fig 6A and 6B show the double-immunocytochemistry assay for NRF-2 (green) and Keap1 [Kelch like-ECH-associated protein, which binds to NRF-2 before its activation] (green), whereas Fig 6C and 6D show the integrated density of each dot using Image J software. As shown in Fig 6A, in absence of stimulus (control cells), low levels of NRF-2 and Keap1 proteins were expressed in the cytoplasm. Whereas the cells exposed to O_3_ (0.1ppm), showed not only an increase in cytoplasmic NRF2 levels, but also substantially greater nuclear translocation, suggesting an internal cellular response to O_3-_induced oxidative stress. When the cells were pre-treated with MIX 1 for 24 hrs, the NRF2 translocation was increased. This is especially noteworthy as the mixture seemed to activate NRF2 without increasing the overall level of oxidative stress, as verified in the previous experiments. Of note, MIX 2 pre-treatment did not significantly affect NRF2 translocation. Furthermore, the level of Keap1 was also measured following O_3_ exposure. As showed in Fig 6B, the expression of Keap1 protein after O_3_ exposure (0.1 and 0.2 ppm) was increased, although not in a dose dependent manner, indicating the potential for a hormetic effect.

### Pre-treatment by antioxidant mixture affects O_3_-induced NF-kB activation

**Fig 7:**
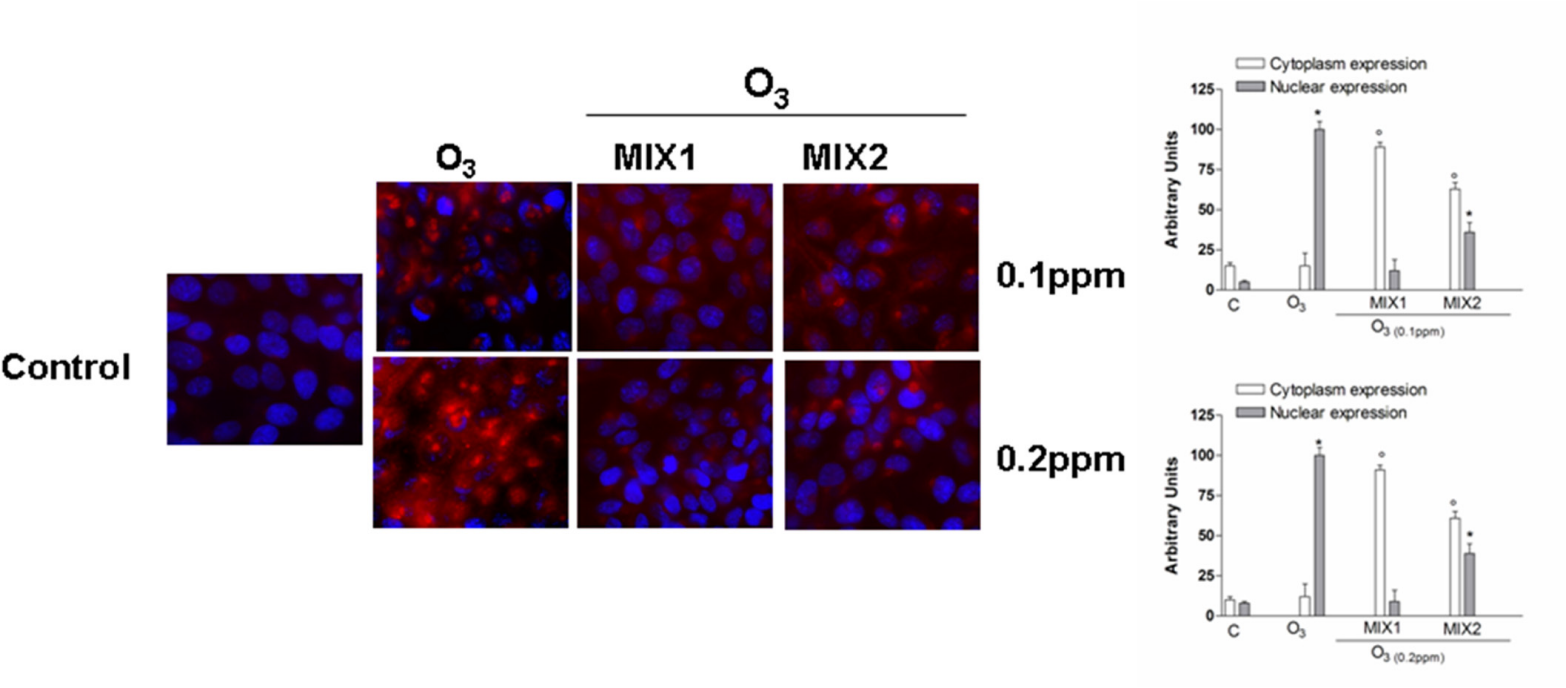
O_3_ induced NF-kB (p65 subunit) activation in human keratinocytes and MIX 1 and MIX 2 pre-treatment for 24 h reverted this effect. Immuno-cytochemistry of keratinocytes showing localization of p65 (red) after O_3_ exposure for 1 h. Images are merged and representative of at least 100 cells viewed in each experiments (n = 5). Nuclei (blue) were stained with DAPI. Original magnification X 630. Immunoreactivity of p65 was semi-quantified as area of activated p65 signal into nucleous respect to that into cytoplasm, by using Image J sophtware. Data are expressed in arbitrary units (averages of five experiments ± SEM, *p < 0.05 vs C (nuclear expression); °*p* < 0.05 vs C (cytoplasm expression)).

Numerous stimuli which cause an accumulation of oxidative stress can lead to the activation of the so-called “redox sensitive transcription factors” and one of the most studies is the nuclear factor kappa-light chain-enhancer of activated B cells (NF-kB) [21]. As showed in Fig 7, in absence of stimulus (control cells), the p65 subunit (red color) was mainly expressed in the cytoplasm. When the cells were exposed to O_3_ (0.1 and 0.2ppm), there was an evident dose dependent increase in p65 nuclear translocation. However, cells pre-treated with either mixture for 24 hrs clearly showed a reduction in NF-kB activation (nuclear translocation) MIX 1 being more effective than MIX 2. Specifically, MIX 1 nuclear expression of NF-kB was undifferentiated from control cells.

**Fig 8:**
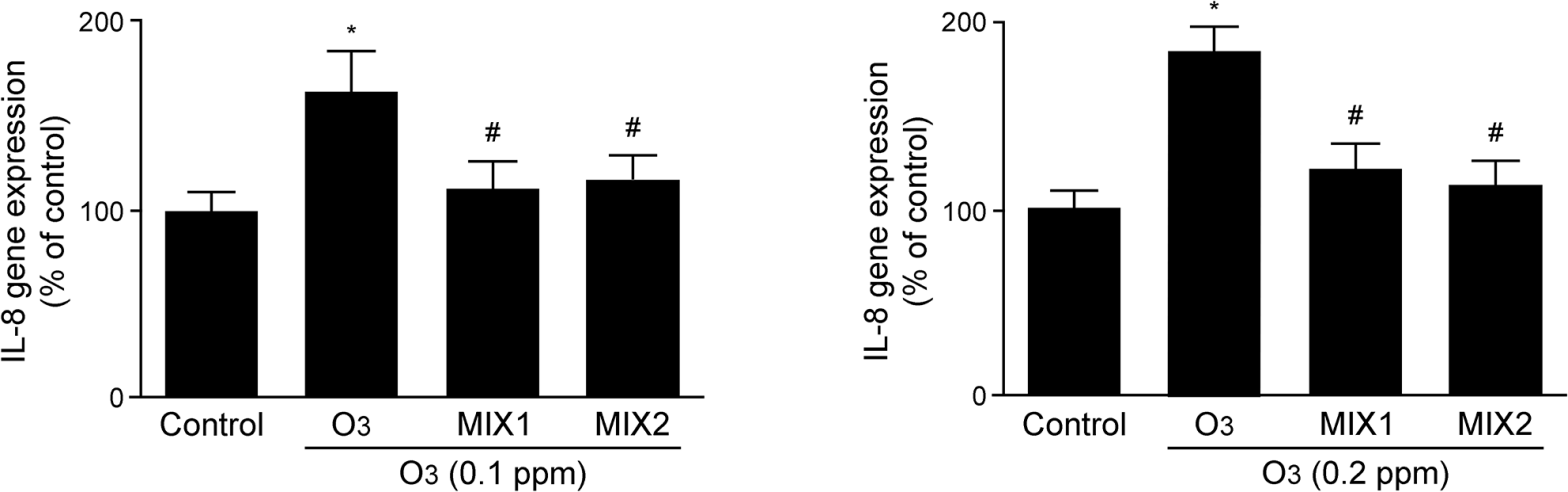
O_3_-induced an increase in IL-8 gene expression in human keratinocytes while MIX 1 and MIX 2 pre-treatment prevented IL-8 induction by O_3_. The data are averages of five different experiments (means ± DS). * p<0.05 vs control; # vs O_3_.

In addition, as a proof of concept for NF-kB activation, the mRNA levels of IL-8, a cytokine under the control of NF-kB, was investigated. As shown in Fig 8, O_3_ exposure clearly induced the increase of IL-8 transcripts in a dose dependent manner with an increment of circa 55% for 0.1 ppm (left panel) and 70% for 0.2 ppm (right panel). Pre-treatments with both MIX 1 and MIX 2 were able to prevent IL-8 induction by O_3_ with a level similar to the control cells.

## Discussion

The present study has shown the ability of antioxidant mixtures containing pure antioxidant compounds to abolish the noxious effects of O_3_ in cultured human keratinocytes. O_3_ is one of the most toxic environmental stressors humans are exposed to on a daily basis, and besides its well-documented effect on the respiratory tracts, there is increasing literature demonstrating its dangerous effect on cutaneous tissue [5, 12, 22, 23]. It should be mentioned that although O_3_ is not considered a radical species per se, its toxic effects ha been shown to be mediated through free radical reactions that leads to the oxidation of biomolecules, and the formation of radical species (hydroxyl radical), with the production of cytotoxic molecules such as aldehydes and more general peroxidation products [23]. While this study offers introductory evidence in support of the protective effect of antioxidants against O_3-_induced damage, further research is needed to confirm these initial findings from cell cultures in more complex models such as skin equivalent and human samples.

The mechanism of O_3_-induced toxicity is believed to be substantially different from other stressors such as UV light [24]. Indeed, it has been clearly shown in the past few decades that UV light, especially UVA, is able to induce skin photodamage primarily through direct formation of ROS, such as O_2_ in the deeper layers of the skin, promoting skin aging and cutaneous neoplasms [25]. On the other hand, the prevailing scientific literature suggests that O_3_ does not penetrate the cellular membrane, and as it concern the skin, reacts instantaneously with polyunsaturated fatty acids (PUFAs) present in the stratum corneum to form ROS, such as hydrogen peroxide and a combination of heterogenous LOPs including HNE [7]. Upon reacting with surface PUFAs, O_3-_induces biochemical changes into deeper tissue by a cascade of ozonation products which further propagate O_3_‘s toxic effect [7]. Additionally, O_3_ oxidizes epidermal antioxidants leading to their depletion [10]. Our study confirmed the formation of lipid peroxidation products and oxidized proteins by O_3_, as measured via HNE protein adducts and protein carbonyls. This effect has previously been demonstrated in an in vivo study [22] where O_3_ exposure induced the formation of HNE. Importantly, the levels of peroxidation products were not homogenously distributed in the skin tissues, but its formation followed a gradient dispersion where higher levels were present in the most external layer (stratum corneum) and less detectable in the dermis. This inversely correlates with the skin antioxidant distribution, where the levels are higher in deeper epidermal layers [23, 26] and lower in the stratum corneum. Therefore, the outermost cellular membranes and their lipids are especially vulnerable targets of O_3_, thus the lipid soluble compounds found in the mixtures utilized in the present study could counteract the noxious effect of O_3_ on epidermal lipids. Most likely the presence of alpha-tocopherol could explain the more potent effect of MIX 1, with respect to MIX 2, in reducing lipid ozonation products.

Throughout the course of evolution, organisms have developed a defence system to protect themselves from the damaging effect of oxidative stress. The cell responds to an increase in ROS prevalence by a rapid induction and activation of detoxifying enzymes in order to minimize further injury [27]. It has been well established that several of the phase II detoxifying enzymes genes contains the so called antioxidant response element (ARE) sequence in their promoter [28] and NRF2 is the main regulator of this cellular defensive mechanism. As mentioned previously, exposure to atmospheric pollutants causes cellular toxicity by promoting a pro-oxidative state, so induction of an antioxidant defense system is a natural way to counteract the damage. Several pathways are able to control cellular redox homeostasis, and NRF2 is a crucial transcription factor in the cell’s response to oxidative stress. Several studies in the last few years have shown the ability of environmental pollutants to activate NRF2, such as particulate matter / nanoparticles, polycyclic aromatic hydrocarbons, and gases such as nitric oxide, carbon monoxide, and O_3_ [27–29]. In the current study, we were able to demonstrate a significant transient induction of NRF2 following O_3_ exposure, while pre-treatment with the tested “mixtures”, resulted in a more robust and longer lasting NRF2 activation leading to greater protection against O_3_-induced oxidative stress. This data is in line with the observed increase in mRNA levels of several genes controlled by NRF2 such as GPx, CAT, HO1, etc by the mixtures (data not shown). This is an especially noteworthy observation since the active compounds comprising the antioxidant mixtures are well established for their ability to reduce the overall oxidative stress level via direct scavenging of ROS [30, 31]. In addition, since NRF2 is able to regulate the transcription of cellular proteasome, its activation will help the cell to eliminate the damaged protein (by-products) accumulated as a consequence of O_3_ exposure (HNE and carbonyls). While MIX 2 was not as effective in prolonging NRF2 activation as MIX 1, both mixtures were still successful in reducing ROS prevalence (as confirmed by DCF-DA assay). For this reason we hypothesize that the beneficial properties of MIX 2 are more linked to direct scavenging of ROS than prolongation of NRF2 activity.

Moreover, pollution and its ability to induce oxidative stress has also been associated with a pro-inflammatory state. Under standard conditions, skin inflammation involves infiltration by neutrophils and additional phagocytes that promote the production of free radical species [32]. Furthermore, there is increasing evidence that keratinocytes serve an intricate role in the pathogenesis of cutaneous inflammatory disease, with earlier literature suggesting that environmental pollutants enhance keratinocyte secretion of pro-inflammatory cytokines (IL-1,IL-8) [33]. Interestingly, the work by Hisada et al. showed that O_3_ exposure induced lung neutrophilia in rats and this response was mainly mediated by the activation of NF-kB, which is able to transcribe for several pro-inflammatory cytokines (IL-1,-8, TNFa, etc) [33]. In line with this study, we have previously shown that O_3_ was able to activate NF-kB in a similar manner as observed in lungs using a murine model [29]. Fundamentally, the activation of NF-kB may be accomplished via accumulation of ROS promoting dissociation of NF-kB from its cytoplasmic repressor IkB [22]. The present study has confirmed the ability of O_3_ to induce NF-kB in keratinocytes and demonstrated the inhibitory effects of the antioxidant mixtures on its activation. This “anti-inflammatory” effect of MIX 1 (containing 15% L-ascorbic acid, 1% alpha-tocopherol, and 0.5% ferulic acid) and MIX 2 (containing 10% L-ascorbic acid, 2% phloretin, and 0.5% ferulic acid) could be attributed to the presence of pure antioxidant compounds able to quench ROS formation, as demonstrated by the DCFH-DA results. Therefore, the tested antioxidant mixtures were not only able to prevent the formation of lipid peroxides, protein oxidation products (HNE and carbonyls), and ROS (DCFH-DA), they also attenuated the activation of NF-kB and transcription of IL-8, key players in modulating the tissue inflammatory response.

It is important to consider that the concentration of ground-level O_3_ has continued to rise over the past decade. A recent work surprisingly showed that there are no significant differences in the percentage of O_3_ increase between urban and rural regions in both the USA and Europe. In fact, O_3_ annual averages continue to increase in both rural and urban areas (albeit at a faster rate in urban centres), exceeding the criteria established to protect human health [34]. As previously mentioned, we have already shown that O_3_ stimulates an active cellular response in the skin [35], and more recently researchers have demonstrated a strong link between O_3_ exposure and an increase in debilitating skin disorders [36]. Therefore, it is imperative to protect our skin from the dangerous effects of O_3_, especially since cutaneous tissue is one of its primary targets. As such, the use of protective antioxidant mixtures with proven ability to neutralize the pro-oxidant effect of pollution is strongly recommended to maintain the healthy integrity of the skin.

## Conclusion

The present study has demonstrated the capacity of antioxidant mixtures to prevent the harmful effects of O_3_-induced oxidative stress in human keratinocytes. In addition to the previously established ability of the tested antioxidant compounds to directly quench free radicals, it was demonstrated that pre-treatment with antioxidant mixtures led to greater activation of NRF2, suggesting complementary protective properties. Moreover, the present study confirmed the ability by O_3_ to induce NF-kB activation in keratinocytes, suggesting the presence of O_3_-induced cutaneous inflammation, and demonstrated the inhibitory effects of the antioxidant mixtures on its activation. These preliminary findings support the benefit of applying pure antioxidants to counteract the noxious effect of O_3_. However, further research is needed to confirm the findings in models more closely resembling human skin.

## References

[1] Health Effects Institute B, MA (2010) HEI Panel on the Health Effects of Traffic-Related Air Pollution. 2010. Traffic-Related Air Pollution: A Critical Review of the Literature on Emissions, Exposure, and Health Effects. HEI Special Report 17.

[2] Sillman S. (1999) The relation between ozone, NOx and hydrocarbons in urban and polluted rural environments. Atmos Environ 33(12):1821–1845.

[3] Mustafa MG. (1999) Biochemical basis of ozone toxicity. Free Radic Biol Med 9(3):245–265. 10.1016/0891-5849(90)90035-h 2272533

[4] Baudouin C, Charveron M, Tarroux R, Gall Y. (2002) Environmental pollutants and skin cancer. Cell Biol Toxicol 18(5):341–348. 12240965 10.1023/a:1019540316060

[5] Valacchi G, van der Vliet A, Schock BC, Okamoto T, Obermuller-Jevic U, Cross CE, et al (2002) Ozone exposure activates oxidative stress response in murine skin. Toxicology 179:163–170. 12204552 10.1016/s0300-483x(02)00240-8

[6] Pryor WA. (1984) Mechanisms of radical formation from reactions of ozone with target molecules in the lung. Free Radic Biol Med 17:451–465. 10.1016/0891-5849(94)90172-4 7835752

[7] Pryor WA, Squadrito GL, Friedman M. (1995) A new mechanism for the toxicity of ozone. Toxicol Lett. 82–83:287–93. 8597067 10.1016/0378-4274(95)03563-x

[8] Thiele JJ, Podda M, Packer L (1997) Tropospheric ozone: An emerging environmental stress to skin. Biol Chem 378:1299–1305. 9426190 10.1515/bchm.1997.378.11.1299

[9] Pryor WA, Church DF. (1991) Aldehydes, hydrogen peroxide, and organic radicals as mediators of ozone toxicity. Free Radic Biol Med 11(1):41–46. 1937128 10.1016/0891-5849(91)90186-7

[10] Thiele JJ, Traber MG, Tsang KG, Cross CE, Packer L. (1997) In vivo exposure to ozone depletes vitamin C and E and induces lipid peroxidation in epidermal layers of murine skin. Free Radical Biol Med 23:85–91. 10.1016/s0891-5849(96)00617-x 9214574

[11] He QC, Tavakkol A, Wietecha K, Begum-Gafur R, Ansari SA, Polefka T. (2006) Effects of environmentally realistic levels of ozone on stratum corneum function. Int J Cosmet Sci 28(5):349–57. 10.1111/j.1467-2494.2006.00347.x 18489299

[12] Valacchi G, Pagnin E, Okamoto T, Corbacho AM, Olano E, Davis PA, et al (2003) Induction of stress proteins and MMP-9 by 0.8 ppm of ozone in murine skin. Biochem Biophys Res Commun 305:741–746. 12763055 10.1016/s0006-291x(03)00812-x

[13] Shindo Y, Witt E, Han D, Epstein W, Packer L. (1994) Enzymic and non-enzymic antioxidants in epidermis and dermis of human skin. J Invest Dermatol 102(1):122–124. 8288904 10.1111/1523-1747.ep12371744

[14] Murray JC, Burch JA, Streilein RD, Iannacchione MA, Hall RP, Pinnell SR. (2007) A topical antioxidant solution containing vitamins C and E stabilized by ferulic acid provides protection for human skin against damage caused by ultraviolet irradiation. J Am Acad Dermatol. 2008 9;59(3):418–25. 10.1016/j.jaad.2008.05.004 18603326

[15] Lin FH, Lin JY, Gupta RD, Tournas JA, Burch JA, Selim MA, et al (2005) Ferulic acid stabilizes a solution of vitamins C and E and doubles its photoprotection of skin. J Invest Dermatol.125:826–832. 16185284 10.1111/j.0022-202X.2005.23768.x

[16] Sticozzi C, Belmonte G, Cervellati F, Di Capua A, Maioli E, Anzini M, et al (2013) Antiproliferative effect of two novel COX-2 inhibitors on human keratinocytes. Eur J Pharm Sci 49:133–41. 10.1016/j.ejps.2013.02.009 23454135

[17] Pecorelli A, Bocci V, Acquaviva A, Belmonte G, Gardi G, Virgili F, et al NRF2 activation is involved in ozonated human serum upregulation of HO-1 in endothelial cells. Toxicol Appl Pharmacol. 2013 2 15;267(1):30–40. 10.1016/j.taap.2012.12.001 23253326

[18] Cervellati F, Pavan B, Lunghi L, Manni E, Fabbri E, Mascoli C, et al Betamethasone, progesterone and RU-486 (mifepristone) exert similar effects on connexin expression in trophoblast-derived HTR-8/SVneo cells. Reprod Fertil Dev. 2011;23(2):319–28. 10.1071/RD10077 21211465

[19] Pryor WA, Squadrito GL, Friedman M. (1995) The cascade mechanism to explain ozone toxicity: the role of lipid ozonation products. Free Radic Biol Med 19(6):935:941. 8582671 10.1016/0891-5849(95)02033-7

[20] Kang KW, Lee SJ, Kim SG. (2005) Molecular mechanism of nrf2 activation by oxidative stress. Antioxid Redox Signal 7(11–12):1664–1673. 16356128 10.1089/ars.2005.7.1664

[21] Bowie A, O'Neill LA. Oxidative stress and nuclear factor-kappaB activation: a reassessment of the evidence in the light of recent discoveries. Biochem Pharmacol. 2000 59(1):13–23. 10605930 10.1016/s0006-2952(99)00296-8

[22] Valacchi G, Pagnin E, Corbacho AM, Olano E, Davis PA, Packer L, et al (2004) In vivo ozone exposure induces antioxidant/stress-related responses in murine lung and skin. Free Radic Biol Med 36: 673–81. 14980710 10.1016/j.freeradbiomed.2003.12.005

[23] Sticozzi C, Valacchi G. (2011) Troposphere ozone as a source of oxidative stress in cutaneous tissue. JSIR 70(11):918–922.

[24] Njus D, Kelley PM. (1991) Vitamin C and E donate single hydrogen atoms in vivo. FEBS Lett 284:147–151 1647978 10.1016/0014-5793(91)80672-p

[25] Farage MA, Miller KW, Elsner P, Maibach HI. (2008) Intrinsic and extrinsic factors in skin ageing: a review. Int J Cosmet Sci. 30:87–95. 10.1111/j.1468-2494.2007.00415.x 18377617

[26] Pryor WA. (1994) Mechanisms of radical formation from reactions of ozone with target molecules in the lung. Free Radic Biol Med 17: 451–65. 7835752 10.1016/0891-5849(94)90172-4

[27] Thiele JJ, Traber MG, Tsang K, Cross CE, Packer L. (1997) In vivo exposure to ozone depletes vitamins C and E and induces lipid peroxidation in epidermal layers of murine skin. FRBM 23:385–391. 10.1016/s0891-5849(96)00617-x 9214574

[28] Weber SU, Thiele JJ, Cross CE, Packer L. (1999) Vitamin C, uric acid, and glutathione gradients in murine stratum corneum and their susceptibility to ozone exposure. J Invest Dermatol 113(6):1128–1132. 10594762 10.1046/j.1523-1747.1999.00789.x

[29] Lee JA, Johnson JA. (2004) An important role of Nrf2-ARE pathway in the cellular defense mechanism. J Biochem Mol Biol 37(2):139–143. 15469687 10.5483/bmbrep.2004.37.2.139

[30] Cho HY, Reddy SP, Kleeberger SR. (2006) Nrf2 defends the lung from oxidative stress. Antioxid Redox Signal 8(1–2):76–87. 16487040 10.1089/ars.2006.8.76

[31] Rubio V, Valverde M, Rojas E. (2010) Effects of atmospheric pollutants on the Nrf2 survival pathway. Environ Sci Pollut Res 17:369–382. 10.1007/s11356-009-0140-6 19367423

[32] Niki E. (1991) Action of ascorbic acid as a scavenger of active and stable oxygen radicals. Am J Clin Nutr 54(6):1119S–1124S 1962557 10.1093/ajcn/54.6.1119s

[33] Ushio H, Nohara K, Fujimaki H. (1999) Effect of environmental pollutants on the production of pro-inflammatory cytokines by normal human dermal keratinocytes. Toxicol Lett 105:17–24 10092052 10.1016/s0378-4274(98)00379-8

[34] Paoletti E, De Marco A, Beddows DC, Harrison RM, Manning WJ. (2014) Ozone and levels in European and USA cities are increasing more than at rural sites, while peak values are decreasing. Environ Pollut 192:295–299. 10.1016/j.envpol.2014.04.040 24906864

[35] Valacchi G, Sticozzi C, Pecorelli A, Cervellati F, Cervellati C, Maioli E. (2012) Cutaneous response to environmental stressors. Ann NY Acad Sci 1271:75–81. 10.1111/j.1749-6632.2012.06724.x 23050967 PMC3495295

[36] Xu F, Yan S, Wu M, Li F, Xu X, Song W, et al (2011) Ambient ozone pollution as a risk factor for skin disorders. Br J Dermatol 165:224–225 10.1111/j.1365-2133.2011.10349.x 21457212

